# Neurophysiologic pain response in patients with juvenlie idiopathic arthritis - a pilot study

**DOI:** 10.1186/1546-0096-12-S1-P147

**Published:** 2014-09-17

**Authors:** Johanne Marie M Iversen, Ellen Dalen Arnstad, Trond Sand, Marite Rygg

**Affiliations:** 1 Department of Laboratory Medicine, Children's and Women's Health, Norwegian University of Science and Technology, Trondheim, Norway; 2 Department of Pediatrics, Hospital of Levanger, Levanger, Norway; 3 Department of Neuroscience, Norwegian University of Science and Technology, Trondheim, Norway; 4 Department of Neurology and Clinical Neurophysiology, University Hospital of Trondheim, Trondheim, Norway; 5 Department of Pediatrics, St. Olavs Hospital, University Hospital of Trondheim, Trondheim, Norway

## Introduction

Pain is a common symptom in children and adolescents with JIA. It has been hypothesized that frequent pain experiences sensitize pain processing pathways, resulting in hypersensitivity to later painful stimuli. A lower pain threshold (PT) and pain tolerance in JIA patients have been demonstrated in previous studies using pressure algometry and the cold pressor task.

## Objectives

To investigate pain thresholds in adolescents with JIA compared to age and sex matched healthy controls, using several modalities for experimental pain testing.

## Methods

Consecutive adolescents with JIA (16-18 years) were recruited from the pediatric rheumatology outpatient clinic at St. Olavs Hospital. Healthy controls were recruited from a local upper secondary school. Both completed a validated questionnaire on health and quality of life (SF-36), and reported pain from the last week (VAS scale). Quantitative sensory testing was conducted, and thermal detection pain thresholds (PTs) recorded. A thermal element was held against three specified locations of the participant’s skin, and the participant was instructed to press a button when he/she felt changes in temperature or pain. Pressure algometry was performed on two well-defined anatomical areas, giving the pressure pain threshold (PPT).

## Results

Compared to 19 healthy controls, the 14 patients with JIA reported more pain during the last week, and had a less favorable score in the physical SF-36 domains, but no difference in the mental health domains. They displayed a lower PPT, but similar cold and warmth PT compared to the controls. When subdividing JIA patients with active and inactive disease, patients with inactive disease had a lower cold PT and PPT, and a tendency towards a lower heat PT compared to controls. Patients with active disease had a tendency towards higher PTs in all three modalities compared to both healthy adolescents and patients with inactive disease.

## Conclusion

Our results indicate that JIA patients may be subject to a sensitization, giving lower pain thresholds in inactive disease, but once the disease is active, painful arthritis may act as a diversion leading to increased rather than lowered PT.

## Disclosure of interest

None declared.

